# Supplementary material for: Optimization Method of the Solvothermal Parameters Using Box–Behnken Experimental Design—The Case Study of ZnO Structural and Catalytic Tailoring
Source: Nanomaterials (Basel). 2021 May 19;11(5):1334. doi: 10.3390/nano11051334 (PMC8159122; doi:10.3390/nano11051334)
Supplement: Supplementary file 1 [file nanomaterials-11-01334-s001.zip › nanomaterials-1208965-supplementary.pdf]

Supplementary Materials

Optimization Method of the Solvothermal Parameters Using Box–Behnken Experimental Design—The Case Study of ZnO Structural and Catalytic Tailoring

Zoltán Kovács, Csanád Molnár, Urška Lavrenčič Štangar, Vasile–Mircea Cristea, Zsolt Pap, Klara Hernadi and Lucian Baia

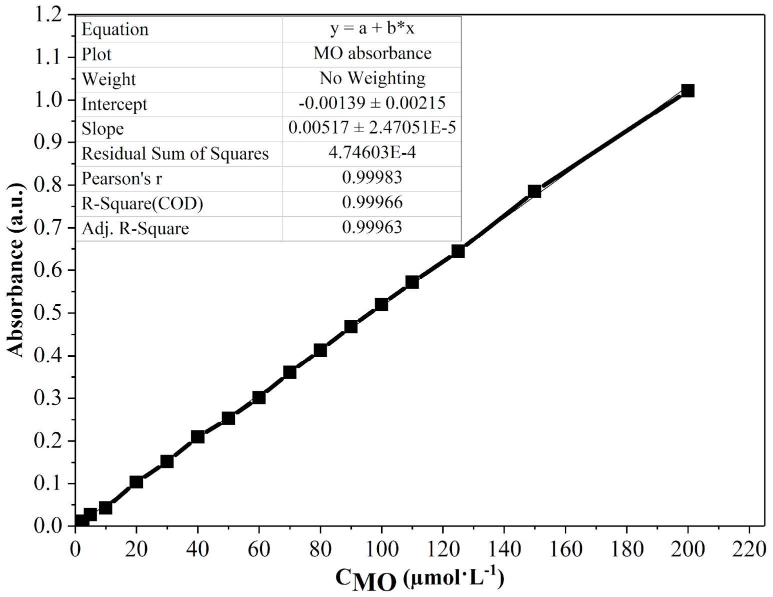

Figure S1. The calibration curve of methyl orange (MO) absorbance at 464 nm at different concentrations (1–200 μM).

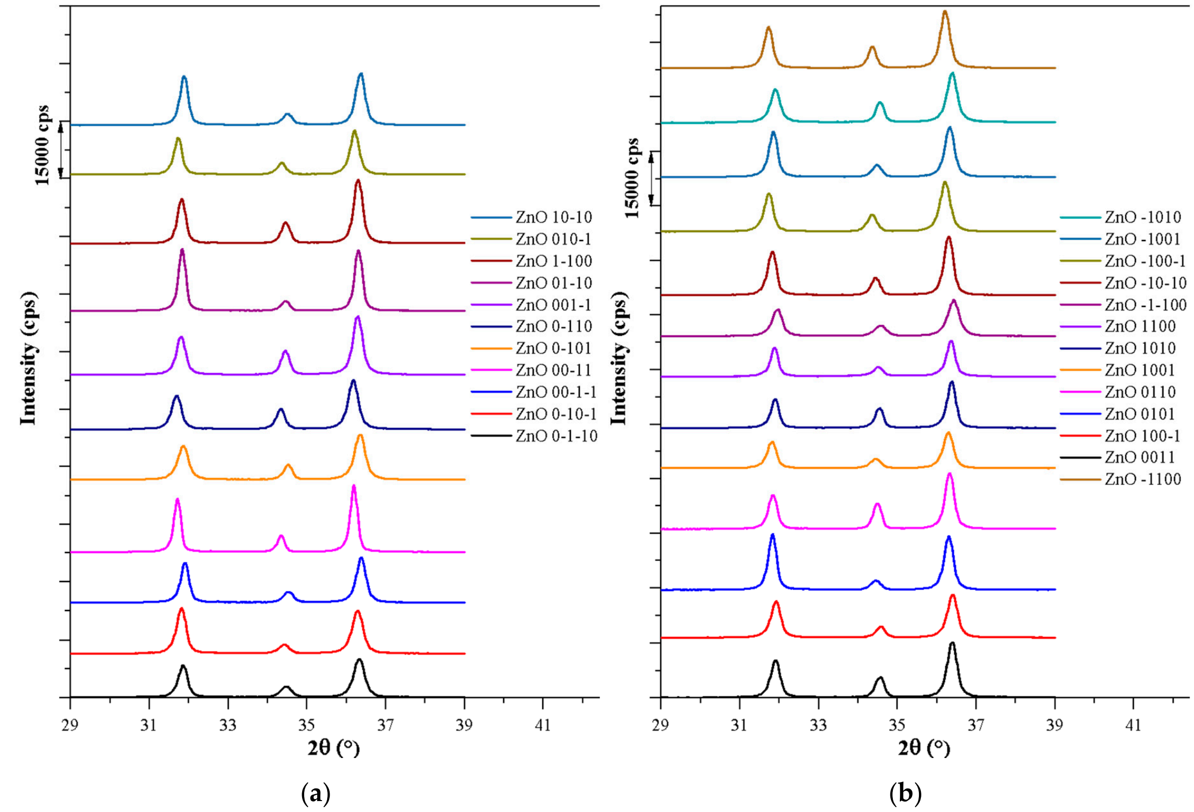

Figure S2. The X-ray diffractograms of all the synthesized samples according to the Box–Behnken Design (BBD) model.

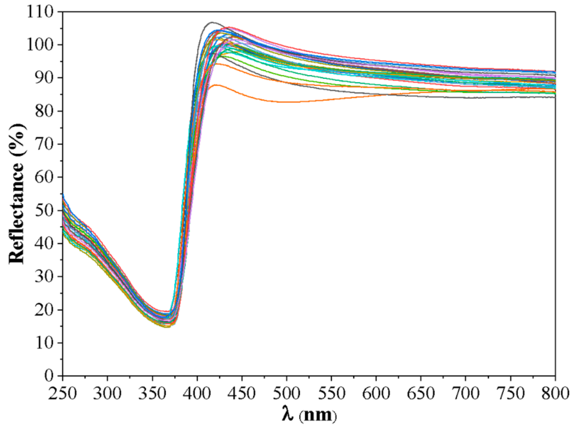

Figure S3. The histograms of residuals of the fitted full quadratic model for photodegradation efficiency (PDE) and $r_{(002)/(100)}$.

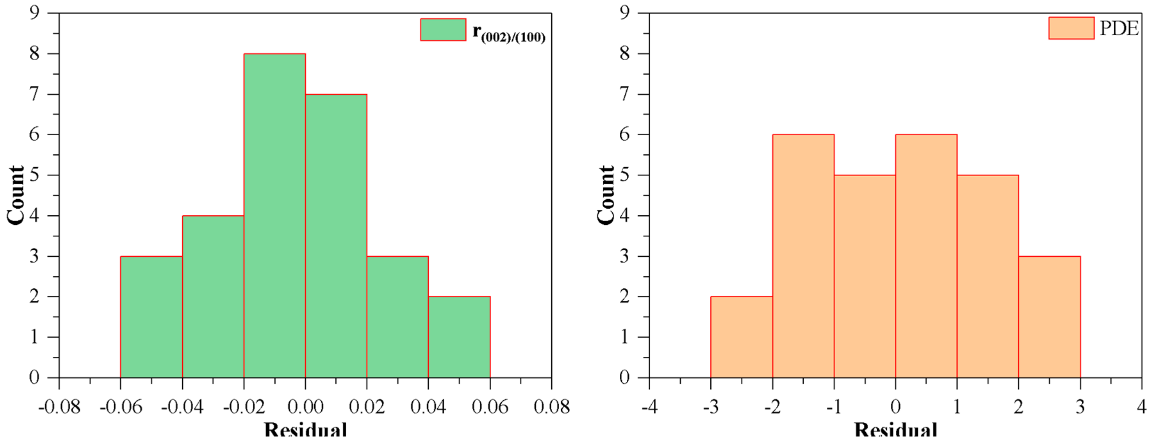

(**a**) (**b**)

Figure S4. The histograms of residuals of the fitted full quadratic model: (**a**) for PDE; (**b**) for $r_{(002)/(100)}$.

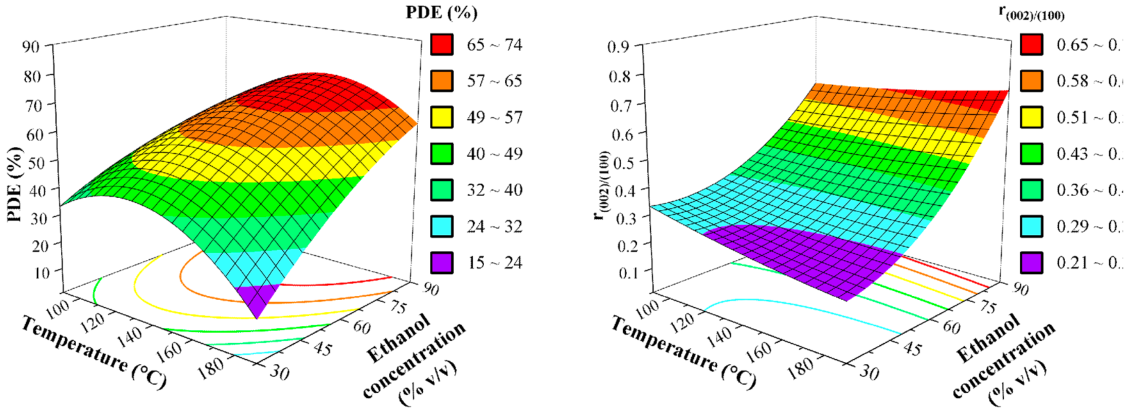

(**a**) (**b**)

Figure S5. Effect of interaction between temperature (X_1) and ethanol concentration (X_3): (**a**) on the PDE; (**b**) $r_{(002)/(100)}$ On the of ZnO as 3D response surface.

Table S1. ANOVA results for quadratic model of ZnO using Box–Behnken design.

Coded Coefficients										
	PDE					$r_{(002)/(100)}$				
**Term**	**Coef**	**SE Coef**	**T-value**	**P-value**	**VIF**	**Coef**	**SE Coef**	**T-value**	**p-value**	**VIF**
Costant	62.27	1.24	50.05	0		0.361	0.023	16.00	0.000	
X_1	−2.142	0.622	−3.44	0.005	1	−0.007	0.011	−0.61	0.551	1
X_2	−4.425	0.622	−7.11	0	1	−0.061	0.011	−5.38	0.000	1
X_3	15.925	0.622	25.6	0	1	0.205	0.011	18.21	0.000	1
X_4	−3.025	0.622	−4.86	0	1	−0.023	0.011	−2.04	0.064	1
X_1^2	−17.046	0.933	−18.27	0	1.25	0.012	0.017	0.72	0.484	1.25
X_2^2	−5.696	0.933	−6.1	0	1.25	0.041	0.017	2.45	0.031	1.25
X_3^2	−5.021	0.933	−5.38	0	1.25	0.106	0.017	6.24	0.000	1.25
X_4^2	−9.521	0.933	−10.2	0	1.25	−0.034	0.017	−2.02	0.066	1.25
$X_1 \times X_2$	−3.42	1.08	−3.18	0.008	1	−0.050	0.020	−2.56	0.025	1
$X_1 \times X_3$	6.62	1.08	6.15	0	1	0.053	0.020	2.73	0.018	1
$X_1 \times X_4$	2.73	1.08	2.53	0.026	1	0.060	0.020	3.07	0.010	1
$X_2 \times X_3$	−13.43	1.08	−12.46	0	1	0.003	0.020	0.14	0.890	1
$X_2 \times X_4$	−3.43	1.08	−3.18	0.008	1	−0.002	0.020	−0.12	0.910	1
$X_3 \times X_4$	7.17	1.08	6.66	0	1	−0.046	0.020	−2.37	0.035	1

Table S2. Experimental runs of Box–Behnken design with the comparison between predicted and experimental photocatalytic degradation efficiency.

Run number	Factors value				PDE	
	X_1	X_2	X_3	X_4		
	°C	M	% *v*/*v*	h	Experimental [%]	Predicted [%]
1	90	0.068	60	8	42.2	42.64
2	90	0.204	60	8	39.3	40.64
3	90	0.136	30	8	31.3	33.00
4	90	0.136	90	8	52.8	51.62
5	90	0.136	60	4	45.5	43.57
6	90	0.136	60	12	32.6	32.05
7	190	0.068	60	8	44.9	45.18
8	190	0.204	60	8	28.3	29.49
9	190	0.136	30	8	13.1	15.45
10	190	0.136	90	8	61.1	60.57
11	190	0.136	60	4	36.3	33.82
12	190	0.136	60	12	34.3	33.20
13	140	0.068	30	8	28.9	26.58
14	140	0.068	90	8	86.7	85.29
15	140	0.068	60	4	48.3	51.05
16	140	0.068	60	12	51.8	51.82
17	140	0.204	30	8	46.2	44.58
18	140	0.204	90	8	50.3	49.59
19	140	0.204	60	4	47.9	49.05
20	140	0.204	60	12	37.7	36.12
21	140	0.136	30	4	42.8	41.96
22	140	0.136	30	12	21.1	21.54
23	140	0.136	90	12	65.3	67.75
24	140	0.136	90	4	58.3	59.48
25	140	0.136	60	8	61.8	62.23
26	140	0.136	60	8	62.1	62.23
27	140	0.136	60	8	62.9	62.23

Table S3. Experimental runs of Box–Behnken design with the comparison between predicted and experimental ratio of intensities of diffraction peaks (0 0 2) and (1 0 0).

Run number	Factors value				PDE	
	X_1	X_2	X_3	X_4		
	°C	M	% *v*/*v*	h	Experimental [%]	Predicted [%]
1	90	0.068	60	8	0.406	0.429
2	90	0.204	60	8	0.444	0.407
3	90	0.136	30	8	0.351	0.332
4	90	0.136	90	8	0.619	0.632
5	90	0.136	60	4	0.429	0.426
6	90	0.136	60	12	0.255	0.259
7	190	0.068	60	8	0.501	0.515
8	190	0.204	60	8	0.339	0.293
9	190	0.136	30	8	0.225	0.213
10	190	0.136	90	8	0.706	0.723
11	190	0.136	60	4	0.292	0.292
12	190	0.136	60	12	0.358	0.365
13	140	0.068	30	8	0.328	0.365
14	140	0.068	90	8	0.802	0.764
15	140	0.068	60	4	0.492	0.446
16	140	0.068	60	12	0.414	0.404
17	140	0.204	30	8	0.197	0.238
18	140	0.204	90	8	0.682	0.648
19	140	0.204	60	4	0.320	0.329
20	140	0.204	60	12	0.233	0.278
21	140	0.136	30	4	0.206	0.203
22	140	0.136	30	12	0.297	0.249
23	140	0.136	90	12	0.582	0.562
24	140	0.136	90	4	0.676	0.700
25	140	0.136	60	8	0.361	0.357
26	140	0.136	60	8	0.371	0.357
27	140	0.136	60	8	0.350	0.357
